# Developmental Programming of Obesity and Liver Metabolism by Maternal Perinatal Nutrition Involves the Melanocortin System

**DOI:** 10.3390/nu9091041

**Published:** 2017-09-20

**Authors:** Paul Cordero, Jiawei Li, Vi Nguyen, Joaquim Pombo, Nuria Maicas, Marco Novelli, Paul D. Taylor, Anne-Maj Samuelsson, Manlio Vinciguerra, Jude A. Oben

**Affiliations:** 1 Institute for Liver and Digestive Health, University College London, London NW3 2PF, UK; jiawei.li.10@ucl.ac.uk (J.L.); v.nguyen@ucl.ac.uk (V.N.); manlio.vinciguerra@fnusa.cz (M.V.); 2 Division of Women’s Health, Faculty of Life Sciences & Medicine, King’s College London, London SE1 7EH, UK; joaquim.1.pombo@kcl.ac.uk (J.P.); nuriamaicas82@gmail.com (N.M.); paul.taylor@kcl.ac.uk (P.D.T.); anne-maj.samuelsson@kcl.ac.uk (A.-M.S.); 3 Department of Pathology, University College London, London WC1E 6JJ, UK; m.novelli@ucl.ac.uk; 4 Center for Translational Medicine, International Clinical Research Center (FNUSA-ICRC), Brno 65691, Czech Republic; 5 Department of Gastroenterology and Hepatology, Guy’s and St Thomas’ Hospital, NHS Foundation Trust, London SE1 7EH, UK

**Keywords:** obesity, developmental programming, Non-Alcoholic Fatty Liver Disease, maternal nutrition, intra-abdominal fat

## Abstract

Maternal obesity predisposes offspring to metabolic dysfunction and Non-Alcoholic Fatty Liver Disease (NAFLD). Melanocortin-4 receptor (Mc4r)-deficient mouse models exhibit obesity during adulthood. Here, we aim to determine the influence of the Mc4r gene on the liver of mice subjected to perinatal diet-induced obesity. Female mice heterozygous for Mc4r fed an obesogenic or a control diet for 5 weeks were mated with heterozygous males, with the same diet continued throughout pregnancy and lactation, generating four offspring groups: control wild type (C_wt), control knockout (C_KO), obese wild type (Ob_wt), and obese knockout (Ob_KO). At 21 days, offspring were genotyped, weaned onto a control diet, and sacrificed at 6 months old. Offspring phenotypic characteristics, plasma biochemical profile, liver histology, and hepatic gene expression were analyzed. Mc4r_ko offspring showed higher body, liver and adipose tissue weights respect to the wild type animals. Histological examination showed mild hepatic steatosis in offspring group C_KO. The expression of hepatic genes involved in regulating inflammation, fibrosis, and immune cell infiltration were upregulated by the absence of the Mc4r gene. These results demonstrate that maternal obesogenic feeding during the perinatal period programs offspring obesity development with involvement of the Mc4r system.

## 1. Introduction

Obesity is a chronic, multifactorial and pro-inflammatory disease defined as a disproportionate increase of body weight with excessive adipose tissue accumulation [1]. The prevalence of obesity is rising alarmingly worldwide, with more than 640 million obese patients and an estimated 1.5 billion overweight people according to the World Health Organization (WHO) [2]. This increase in adiposity is associated with all causes of mortality, a significant decrease in lifespan of up to 20 years, and a tremendous fiscal burden [3,4]. Obesity is associated with multiple comorbidities representing the main causes of illness and death in affluent societies, especially cardiovascular and cerebrovascular illnesses, type 2 diabetes mellitus, many cancers, and Non-Alcoholic Fatty Liver Disease (NAFLD) [1,5]. NAFLD is now the most common cause of liver disease in these affluent countries; it may progress through steatosis, inflammation and injury (non-alcoholic steatohepatitis, NASH), fibrosis, cirrhosis, and hepatocellular carcinoma [6,7,8]. Considering that the prevalence of obesity and NAFLD in Western countries ranges between 20–30%, these alterations in liver morphology and functionality secondary to NAFLD are a major concern for national health policies [6].

The increase in global obesity rate affects all populations, including women in their reproductive age. As a result, the risk of pregnancy loss, maternal gestational diabetes, fetal malformations, and other complications during pregnancy has increased in obese women [9]. Interestingly, retrospective epidemiological human studies and animal interventions have recently highlighted that, during early development, an adverse pro-obesogenic in utero environment plays an important role in promoting offspring obesity and metabolic diseases in later life [10]. Our previous studies have demonstrated that maternal obesogenic diet during perinatal periods programs the development of obesity and NAFLD in the offspring [11,12,13], although the precise involved mechanism remains uncertain.

The etiology of obesity is mostly thought of, perhaps simplistically, as higher caloric intake greater than energy expenditure. However, the underlying mechanisms are much more complex and include genetic predisposition, epigenetic regulation, environmental factors, and/or interactions with the gut microbiota [1,14]. Indeed, current Genome Wide Association Studies (GWAS) point to several key genes with very important influences on the origin and development of obesity: these include Fat-Associated Obesity (FTO), Leptin, Leptin Receptor, Pro-Opiomelanocortin (Pomc), or Melanocortin Receptor 4 (Mc4r) [15]. Importantly, multiple meta-analyses and GWAS studies have confirmed the association between Mc4r polymorphisms and obesity and its associated comorbidities [16,17,18]. Mc4r is a critical mediator in energy homeostasis, regulating both food intake and energy expenditure as well as affecting blood pressure homeostasis [19,20]. Interestingly, a novel study in rats by Tabachnik et al. demonstrated that perinatal obesogenic environment increased in the offspring histone acetylation marks at the Mc4r promoter. This epigenetic regulation was also associated with thyroid hormones metabolism as well as with the inhibition of Mc4r transcription [21]. The aim of this study, therefore, was to investigate ab initio whether the Mc4r gene plays a role in the maternal programming of offspring obesity and consequent NAFLD.

## 2. Materials and Methods

### 2.1. Animals and Experimental Design

All experiments were approved by the Local Ethics Committee of the University of King’s College London, and were conducted in accordance with the Home Office Animals (Scientific Procedures) Act of 1986 guidelines (United Kingdom). Mice were housed under controlled conditions (light-dark cycle 12 h, 21 ± 2 °C, 40–50% humidity) with food and water available ad libitum. Adult female mice heterozygous for Mc4r with C57BL/6J background were fed an obesogenic diet (824053, Special Dietary Services, Wittam, UK) [22] supplemented with sweetened condensed milk (Nestlé, Vevey, Switzerland) and fortified with 3.5% (AIN 93G; Special Diets Services) mineral mix and 1% vitamin mix or a control standard laboratory diet (RM1, Special Diets Services) for 5 weeks (dietary composition in Table 1). Then, as previously described, obesogenic-fed heterozygous females were around 50% heavier than control-fed females [23]. The female mice were mated with control-fed heterozygous males from the same litter. Conception was determined by vaginal plug formation. The female animals were maintained on their allocated diets throughout gestation and lactation, as previously described [11]. Litter sizes from both maternal feeding groups were similar [23]. After birth, litters were standardized to six pups each with an equal number of males and females when possible. At day 21 postnatally, offspring were genotyped and weaned onto a control diet until 6 months old. They were then killed by schedule 1 method after an overnight fast. Blood samples were collected, centrifuged (10,000× *g*, 10 min at 4 °C), and stored at −80 °C until further analysis. Liver and inguinal adipose depots were harvested, weighted, and stored at −80 °C. A representative sample of each liver was fixed in 10% formalin for histological analysis.

### 2.2. Liver Histology

Offspring liver samples at 6 months of age (*n* = 5–6 per experiment group) were fixed in formalin (10%), dehydrated, and subsequently embedded in paraffin. Liver samples were cut into 4-μm sections, mounted, and dried overnight at 37 °C. The liver sections were then stained with hematoxylin and eosin (H&E), and the extent of steatosis assessed by an expert liver pathologist blinded to the group identities, as previously described [24].

### 2.3. Plasma Analysis

Plasma glucose, triglycerides, alanine aminotransferase (ALT), and aspartate aminotransferase (ALT) concentrations were assayed by the Royal Free Hospital Clinical Biochemistry Department (London, UK).

### 2.4. mRNA Extraction and Real-Time qPCR

Frozen liver samples (*n* = 5–6 per experiment group) were homogenized using TRIzol Reagent (Invitrogen, Carlsbad, CA, USA) and mRNA was extracted by following the suppliers’ protocol. Sample quality and concentrations were measured using a NanoDrop ND-1000 Spectrometer (Thermo Scientific, Waltham, MA, USA). DNase treatment and retrotranscription to cDNA were carried out using the Qiagen QuantiTect Reverse Transcriptase kit (Qiagen, Hilden, Germany). Quantitative real-time PCR (rt-qPCR) was performed by triplicate using the ABI PRISM 7500 HT Fast real-time PCR system (Applied Biosystems, Austin, TX, USA) with QuantiFast SYBR Green PCR (Qiagen, Hilden, Germany). 18S was used as a control for cDNA quality and Gapdh was used as the control housekeeping reference gene. All designed primers were obtained from Sigma-Aldrich (St. Louis, MO, USA) and a melting curve analysis confirmed the amplification of specific PCR products and the absence of non-specific amplification or primer-dimers. Gene-specific primer sequences for 18S ribosomal RNA (18S), glyceraldehyde-3-phosphate dehydrogenase (Gapdh), alpha-smooth muscle actin (α-SMA), tumor necrosis factor alpha (TNF-α), collagen type I alpha 1 (Col-1α), interleukin 6 (IL6), chemokine (C-C motif) ligand 2 (MCP1), interleukin 1 beta (IL-1β), and transforming growth factor beta (TGF-β) are listed in Table 2. Fold changes between groups were calculated using the 2^−ΔΔct^ method.

### 2.5. Statistical Analysis

All data are expressed as the mean ± standard error of the mean (SEM). Two-way ANOVA was applied for studying the effect of maternal obesogenic feeding (C vs. Ob) and offspring genotype (wt vs. knockout). Comparison of the means was carried out by Tukey post-hoc test. The statistical unit used throughout the analysis was the number of dams. Statistical significance was accepted with a *p* value of less than 0.05. IBM SPSS 24 software (24.0, SPSS Statistics, IBM, Chicago, IL, USA) was used for the statistical analysis.

## 3. Results

### 3.1. Phenotypic and Histological Characteristics

**Figure 1:**
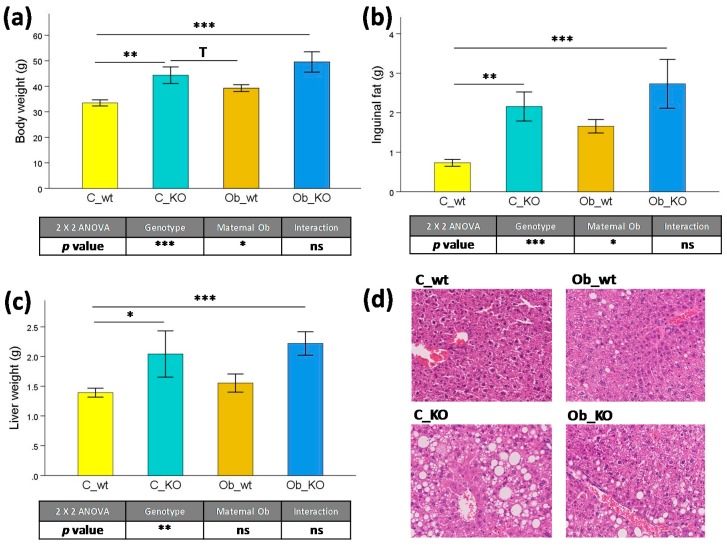
Phenotypic and histological parameters. Effects of Mc4r gene deletion and maternal obesogenic feeding on (**a**) body weight, (**b**) inguinal fat weight, (**c**) liver weight as well as (**d**) hepatic representative histological Hematoxylin and Eosin stained sections. Mc4r, melanocortin 4 receptor; C_wt, control wild type; C_KO, control knockout; Ob_wt, obese wild type; Ob_KO, obese knockout; n.s., non-significant; * *p* < 0.05; ** *p* < 0.01; *** *p* < 0.001; T *p* > 0.05 and *p* < 0.1.

We firstly analyzed the effect of maternal obesogenic feeding on phenotypical parameters and hepatic morphology (Figure 1). As we have previously reported, at 6 months of age, body weight of Mc4rko and wild type mice from control- and obesogenic-fed dams had already reached a plateau [23]. Thus, at this age, there was a marked genotype effect independent of maternal nutrition, with increased body mass (+0.37-fold, *p* < 0.001) (Figure 1a), inguinal fat mass (+1.59-fold, *p* < 0.001) (Figure 1b), and liver weight (+1.51-fold, *p* < 0.01) (Figure 1c) in KO mice compared to the wild type animals. Furthermore, maternal obesogenic feeding during perinatal periods predisposed the offspring to higher body weight (+0.27-fold, *p* < 0.05) and inguinal fat deposition (+1.19-fold, *p* < 0.05). In offspring subjected to maternal control diet, C_KO mice presented a marked obesity phenotype compared to C_wt animals, with higher body mass (+0.29-fold, *p* < 0.01), inguinal fat mass (+1.96-fold, *p* < 0.01), and liver weight (+0.47-fold, *p* < 0.05). Finally, the combination of maternal obesity and Mc4r gene deletion strongly influenced offspring phenotype when compared to the C_wt group, showing a marked increase in body mass (+0.48-fold, *p* < 0.001), inguinal fat mass (+2.47-fold, *p* < 0.001), and liver weight (+0.60-fold, *p* < 0.001). According to the hepatic morphology (Figure 1d), there was a mild steatosis in C_KO animals with no changes in the general liver architecture.

### 3.2. Plasma Biochemical Features

**Figure 2:**
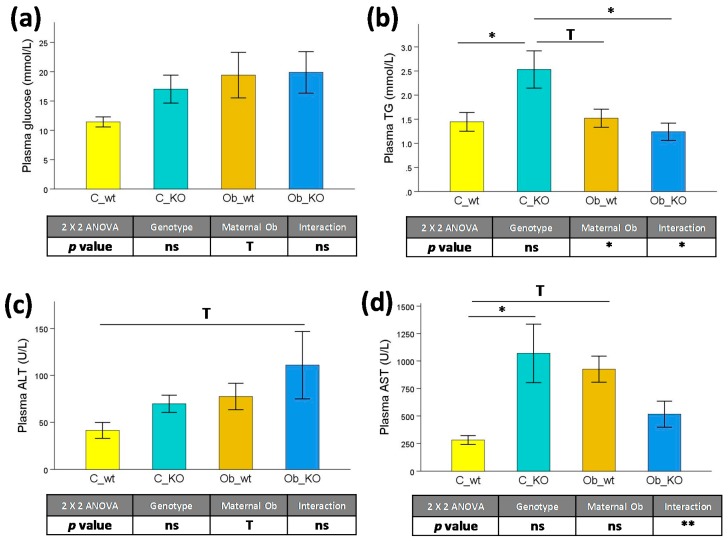
Plasma biochemical profile. Effects of Mc4r gene deletion and maternal obesogenic feeding on (**a**) Glucose, (**b**) Triglycerides, (**c**) ALT, and (**d**) AST concentrations. Mc4r, melanocortin 4 receptor; C_wt, control wild type; C_KO, control knockout; Ob_wt, obese wild type; Ob_KO, obese knockout; n.s., non-significant; * *p* < 0.05; T *p* > 0.05 and *p* < 0.1; TG: triglycerides; ALT: alanine aminotransferase; AST: aspartate aminotransferase.

Plasma glucose concentration (Figure 2a) showed a tendency to be increased (+0.38-fold, *p* < 0.1) in the offspring subjected to maternal obesity compared to control-fed dams. However, the absence of the Mc4r gene had no effect on this parameter. Furthermore, there was a decrease in plasma triglyceride concentration (Figure 2b) in offspring from obese mothers (−0.30-fold, *p* < 0.05) compared to the controls. However, this effect was mainly caused by the elevated TG levels in the C_KO group compared to the C_wt (+0.75-fold, *p* < 0.05), Ob_wt (+0.67-fold, *p* < 0.1), and Ob_KO groups (+1.05-fold, *p* < 0.05). With the hepatic transaminases (Figure 2c,d), there was a trend of increased ALT caused by maternal obesity (+0.74-fold, *p* < 0.1), which may be explained by the elevated concentrations of this transaminase in the Ob_KO with respect to the C_wt group (+1.68-fold, *p* < 0.1). Additionally, AST was markedly increased by the absence of the Mc4r gene in offspring from control-fed dams (+0.40-fold, *p* < 0.05) and partially increased in wild type animals subjected to maternal obesogenic feeding (+2.80-fold, *p* < 0.1).

### 3.3. Hepatic Transcriptomic Profile

**Figure 3:**
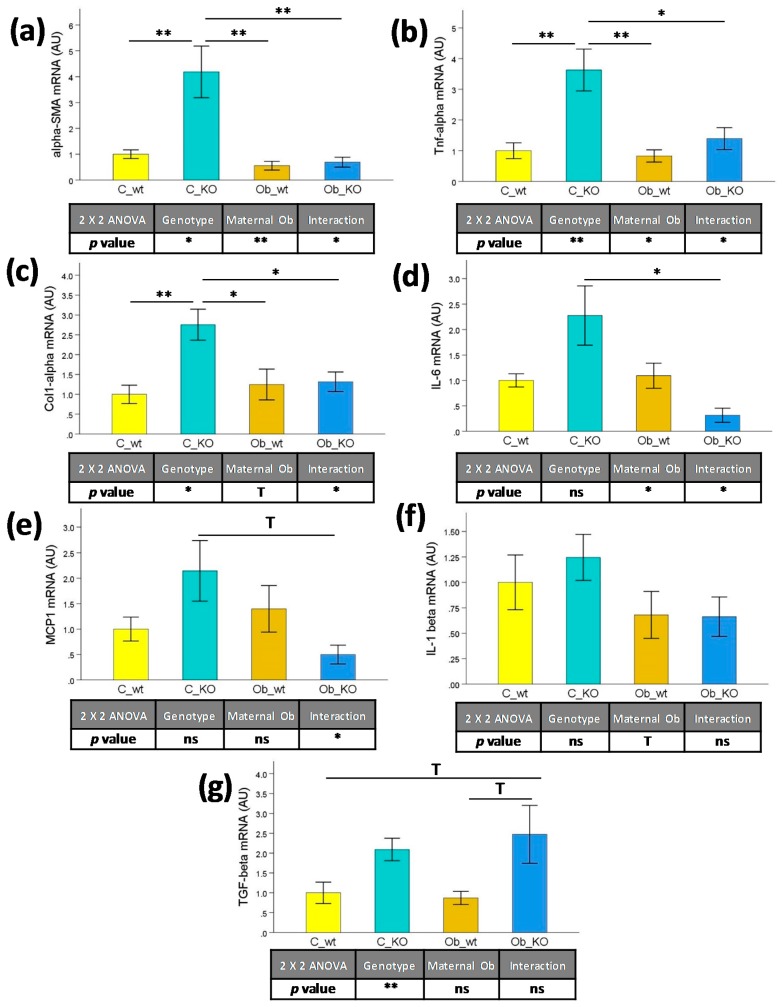
Liver mRNA levels by real-time qPCR. Effects of Mc4r gene deletion and maternal obesogenic feeding on (**a**) α-SMA, (**b**) TNF-α, (**c**) Col-1α, (**d**) IL6, (**e**) MCP1, (**f**) IL-1β, and (**g**) TGF-β expression. Mc4r, melanocortin 4 receptor; C_wt, control wild type; C_KO, control knockout; Ob_wt, obese wild type; Ob_KO, obese knockout; n.s., non-significant; * *p* < 0.05; ** *p* < 0.01; *** *p* < 0.001; T *p* > 0.05 and *p* < 0.1; α-SMA: alpha-smooth muscle actin; TNF-α: tumor necrosis factor alpha; Col-1α: collagen type I alpha 1; IL6: interleukin 6; MCP1: chemokine (C-C motif) ligand 2; IL-1β: interleukin 1 beta; TGF-β: transforming growth factor beta; AU: arbitrary units.

We also studied the effect of maternal and obesogenic feeding on hepatic mRNA expression of genes associated with inflammation and immune mediation. As depicted in Figure 3, there was a marked effect of the genotype on the expression of α-SMA (+2.33-fold, *p <* 0.05) (Figure 3a), TNF-α (+1.85-fold, *p* < 0.01) (Figure 3b), Col-1α (+0.90-fold, *p <* 0.05) (Figure 3c), and TGF-β (+1.36-fold, *p* < 0.01) (Figure 3g) in KO mice independent of maternal feeding. On the other hand, maternal obesity compounded with the Mc4r KO genotype had decreased α-SMA (−0.73-fold, *p* < 0.01), TNF-α (−0.54-fold, *p* < 0.05), and IL6 (−0.56-fold, *p* < 0.05) (Figure 3d) hepatic mRNA levels in the offspring. These effects were mainly explained by the characteristic transcriptional profile of the C_KO group, which showed a consistent higher expression of α-SMA (from +3.19-fold to +6.61-fold, *p* < 0.01), TNF-α (from +1.59-fold to +3.37-fold, from *p* < 0.05 to *p* < 0.01,) and Col-1α (from +1.08-fold to +1.75-fold, from *p* < 0.05 to *p* < 0.01) with respect to the C_wt, Ob_wt, and Ob_KO groups. 

## 4. Discussion

Observations of human polymorphisms highlight the Mc4r gene as one of the key genes for understanding obesity risk and its associated comorbidities [16,17,18]. Mc4r is shown to be an energy balance modulator. Recently, a mouse study described that the activation of Mc4r reduces food intake and increases energy expenditure, preventing obesity-associated increased adiposity [25]. Additionally, the absence of Mc4r inhibits brown adipose tissue activity; therefore, the stimulation of the Mc4r pathway can be a potential target for increasing energy expenditure and accelerating weight loss [26]. Although melanocortin receptors are predominantly expressed in the brain, Mc4r is also known to be present in liver cells [27,28]. Therefore, the lack of this gene not only exerts systemic effects through the nervous system, but may also have a direct hepatic component. Evidence from liver regeneration after acute liver injury, where rats were subjected to partial hepatectomy, has shown that there is an overexpression of Mc4r in the hepatocytes [29]. Furthermore, NAFLD is the main hepatic manifestation of the metabolic syndrome, often accompanied by alterations in glucose homeostasis and waist circumference, and has been directly associated with genetic variations of Mc4r [30].

Itoh et al. reported that Mc4r_KO mice developed steatohepatitis when fed a high-fat diet, which was associated with an obese phenotype, insulin resistance, and dyslipidemia. Histologic analysis found enhanced inflammation, macrophage infiltration, hepatocyte ballooning, and, after a year of obesogenic feeding, hepatocellular carcinoma [31]. However, these results should be carefully compared with our experimental model because the direct, long-term effects of adult obesogenic feeding have greater impact on mice metabolism than maternally induced obesity. Probably due to this reason, our liver phenotypes did not present as marked of a proinflammatory stage. In the previous study, the authors also described obesity-related traits in Mc4r-silenced mice fed a control diet; similar to what we have showed here in our study, there was overexpression of TGF-β and Col-1α compared to wild type mice [31]. In vitro studies have also shown that the treatment of isolated liver cells with melanocortin agonists inhibits endotoxin-induced upregulation of the pro-inflammatory cytokines IL-6, IL1β, and TNF-α by Kupffer cells [28]. Thus, changes we described in liver gene expression in our Mcr4_ko offspring from control-fed dams may be the initial step for the apparition of later fibrotic markers in the liver, in addition to the detection of infiltrated macrophages and their polarization to different subpopulations. Indeed, there was a tendency to increase Mcp1 hepatic expression in these animals, which in turn may exacerbate, as we have shown, the hepatic expression of pro-inflammatory and immune system-related genes.

Maternal perinatal physiology and environmental insults predispose offspring to metabolic diseases in adult life. Thus, our previous studies with rodent models have demonstrated that a hypercaloric diet enriched in fat and simple sugars during peri-conception, pregnancy, and/or lactation periods affects offspring phenotype with increased body weights, visceral fat, liver and pancreas weights, plus a parallel accumulation of lipids in visceral organs [11,12,13,32]. Our previous results showed that maternal obesity programs development of a dysmetabolic and NAFLD phenotype, which is critically dependent on the early postnatal period involving alteration of hypothalamic appetite nuclei signaling by maternal breast milk and neonatal adipose tissue-derived leptin [12,32]. Furthermore, in a perinatal model of mice lacking the Mc4r gene, we demonstrated that maternal obesity (apparently through neonatal leptin exposure) permanently resets the responsiveness of the central sympathetic nervous system, specifically via the hypothalamic paraventricular nucleus melanocortin system, to initiate hypertension [23]. Moreover, in that study, we found increased food intake and leptin plasma levels influenced by maternal obesity and by the lack of the Mc4r gene. Surprisingly, in the current study, we found that the offspring phenotype was more influenced by the lack of the Mc4r gene, rather than by maternal obesity. Indeed, although maternal obesogenic feeding was associated with higher body and adipose depots, there was a lack of steatosis in liver histological samples. This may be partially explained by the age of these animals, as in our previous murine studies with similar feeding protocol, the steatotic effect induced by maternal obesity was well defined at 12 months and vague at 6 months of age [12,33]. Indeed, the age of these animals is directly proportional to their intra-abdominal adipose tissue accumulation and, therefore, to the abnormal fat infiltration in visceral organs. Interestingly, we did not find an additional effect of the lack of Mc4r on the maternal obesogenic feeding offspring. We may hypothesize that the molecular mechanisms affecting obesity and the associated liver fat accumulation and damage may be common for maternal-associated programming of obesity and for Mc4r pathways. For example, there is appetite regulation in both situations as well as a decrease in energy expenditure induced by maternal obesity and Mc4r blockage [23,26]. Moreover, a study in rats with high-fat diet-induced maternal obesity recently described a downregulation of hypothalamic Mc4r mRNA expression at weaning in the offspring from obese dams [34]. Others have replicated these results, proposing an epigenetic mechanism for the decrease in Mc4r expression in the offspring of obese rats due to histone acetylation in the Mc4r promoter region, which may also be associated with the thyroid hormone receptor-β, a transcription inhibitor for this gene [21]. This research group has also described how other Mc4r-related genes involved in obesity through appetite regulation such as Pomc may be epigenetically regulated in the offspring because of maternal obesity [35,36].

As a limitation of this study, the use of animal models and, more specifically, knockout and perinatally-based designs makes the translation of the findings to the general population difficult. However, besides the ethical considerations of human interventions during pregnancy, rodent models shorten the experimental time, and also allow studying the effects during offspring adult life. Furthermore, the similar genetic and physiological background to humans and the control of external insults and confounding factors make necessary to perform experimental animal models in this field. Furthermore, although phenotypically the offspring were influenced by maternal obesity, from a metabolic and transcriptomic point of view the effect became partially diluted, which differs with our previously standardized developmental programing protocols [11,12,13,33,37]. This may be due to the Mc4r gene silencing; however, the lack of difference in some of the variables only due to maternal obesogenic feeding may be also due to a limited number of animals and the wide intra group differences. Finally, the lack of some interesting plasma and hepatic biochemical markers such as liver triglyceride content or food consumption may be a limitation for the explanation of the findings described in the current study.

## 5. Conclusions

In conclusion, these results emphasized the importance of the melanocortin system as a target for the development of new therapeutic tools against obesity and its associated implications in liver metabolism through obesogenic feeding and developmental programming. We showed that dietary changes during the perinatal period may follow an adaptive response of the offspring to be predisposed to long-term changes in metabolism and physiology. Although the lack of Mc4r induced an increase in body, fat, and liver weights, the interaction with maternal perinatal obesity suggested a protective effect in the Mc4r_ko mice. Thus, offspring from obese mothers did not show liver steatosis and presented lower hepatic expression of proinflammatory and profibrogenic genes. This interaction should warrant further research in this model, given the potential to elucidate new mechanistic pathways implicated in the developmental programing of obesity and NAFLD.

## Figures and Tables

**Table 1:** Macronutrient composition of the diets.

Dietary Composition (g/Kg)	Control	Obesogenic	Condensed Milk
Protein	144	230	80
Amino Acids			
Glutamic Acid	31.7	45.5	16.6
Proline	12	24.8	7.7
Leucine	9.8	20.5	7.8
Aspartic Acid	6.7	15.4	6
Serine	5.6	12.9	4.3
Valine	6.9	14.5	5.3
Lysine	6.6	18.9	6.3
Glycine	11.1	4.1	1.7
Arginine	9.1	8.1	2.9
Others	44.5	65.3	20.5
Carbohydrates			
Polysaccharides	500	283	0
Simple sugars	40	105	550
Cellulose	43.2	61.7	
Hemicellulose	101.7		
Lipid	27	226	90
Saturated Fatty Acids	5.1	76.2	59.4
Monounsaturated Fatty Acids	8.8	85.2	24.3
Polyunsaturated Fatty Acids	8.8	39.1	3.4
Mineral content	35		
Vitamin content	4.1		
AIN-93G mineral mix			1.68
AIN-93M mineral mix		43	
Vitamin mix		12	
Energy (kcal/g)	3.52	4.54	3.22

**Table 2:** Rt-qPCR primer sequences.

Gene	Primer Sequence
18S	sense: AGTCCCTGCCCTTTGTACACA
	antisense: CGATCCGAGGGCCTCACTA
Gapdh	sense: CGTCCCGTAGACAAAATGGT
	antisense: TCAATGAAGGGGTCGTTGAT
α-SMA	sense: CTCTTGCTCTGGGCTTCATC
	antisense: GGCTGTTTTCCCATCCATC
TNF-α	sense: CCACCACGCTCTTCTGTCTA
	antisense: AGGGTCTGGGCCATAGAACT
Col-1α	sense: GTCCCCGAGGCAGAGATG
	antisense: GTCCAGGGCCAGATGAAACT
IL6	sense: TCAATTCCAGAAACCGCTATG
	antisense: GTCTCCTCTCCGGACTTGTG
MCP1	sense: CCCACTCACCTGCTGCTACT
	antisense: TCTGGACCCATTCCTTCTTG
IL-1β	sense: CAACCAACAAGTGATATTCTCCATG
	antisense: GATCCACACTCTCCAGCTGCA
TGF-β	sense: AAAATCAAGTGTGGAGCAAC
	antisense: CCACGTGGAGTTTGTTATCT

18S: 18S ribosomal RNA; Gapdh: glyceraldehyde-3-phosphate dehydrogenase; α-SMA: alpha-smooth muscle actin; TNF-α: tumor necrosis factor alpha; Col-1α: collagen type I alpha 1; IL6: interleukin 6; MCP1: chemokine (C-C motif) ligand 2; IL-1β: interleukin 1 beta; TGF-β: transforming growth factor beta.
